# Fine mapping of genetic susceptibility loci for melanoma reveals a mixture of single variant and multiple variant regions

**DOI:** 10.1002/ijc.29099

**Published:** 2014-07-31

**Authors:** Jennifer H Barrett, John C Taylor, Chloe Bright, Mark Harland, Alison M Dunning, Lars A Akslen, Per A Andresen, Marie-Françoise Avril, Esther Azizi, Giovanna Bianchi Scarrà, Myriam Brossard, Kevin M Brown, Tadeusz Dębniak, David E Elder, Eitan Friedman, Paola Ghiorzo, Elizabeth M Gillanders, Nelleke A Gruis, Johan Hansson, Per Helsing, Marko Hočevar, Veronica Höiom, Christian Ingvar, Maria Teresa Landi, Julie Lang, G Mark Lathrop, Jan Lubiński, Rona M Mackie, Anders Molven, Srdjan Novaković, Håkan Olsson, Susana Puig, Joan Anton Puig-Butille, Nienke van der Stoep, Remco van Doorn, Wilbert van Workum, Alisa M Goldstein, Peter A Kanetsky, Paul D P Pharoah, Florence Demenais, Nicholas K Hayward, Julia A Newton Bishop, D Timothy Bishop, Mark M Iles

**Affiliations:** 1 Section of Epidemiology and Biostatistics, Leeds Institute of Cancer and Pathology, University of LeedsLeeds, United Kingdom; 2 Department of Oncology, University of CambridgeCambridge, United Kingdom; 3 Centre for Cancer Biomarkers CCBIO, Department of Clinical Medicine, University of BergenBergen, Norway; 4 Department of Pathology, Haukeland University HospitalBergen, Norway; 5 Department of Pathology, Molecular Pathology, Oslo University HospitalRikshospitalet, Oslo, Norway; 6 Assistance Publique–Hôpitaux de Paris, Hôpital Cochin, Service de Dermatologie, Université Paris DescartesParis, France; 7 Department of Dermatology, Sheba Medical Center, Tel Hashomer, Sackler Faculty of MedicineTel Aviv, Israel; 8 Oncogenetics Unit, Sheba Medical Center, Tel Hashomer, Sackler Faculty of Medicine, Tel Aviv UniversityTel Aviv, Israel; 9 Department of Internal Medicine and Medical Specialties, University of GenoaGenoa, Italy; 10 Laboratory of Genetics of Rare Hereditary Cancers, IRCCS AOU San Martino-ISTGenoa, Italy; 11 INSERM, UMR-946, Genetic Variation and Human Diseases UnitParis, France; 12 Université Paris Diderot, Sorbonne Paris Cité, Institut Universitaire d'HématologieParis, France; 13 Division of Cancer Epidemiology and Genetics, National Cancer Institute, National Institutes of HealthBethesda, MD; 14 International Hereditary Cancer Center, Pomeranian Medical UniversitySzczecin, Poland; 15 Department of Pathology and Laboratory Medicine, Perelman School of Medicine at the University of PennsylvaniaPhiladelphia, PA; 16 Epidemiology and Genomics Research Program, Division of Cancer control and Population Sciences, National Human Genome Research Institute, National Institutes of HealthBaltimore, MD; 17 Department of Dermatology, Leiden University Medical CentreLeiden, The Netherlands; 18 Department of Oncology-Pathology, Karolinska Institutet, Karolinska University HospitalSolna, S-171 76, Stockholm, Sweden; 19 Department of Dermatology, Oslo University HospitalRikshospitalet, N-0027, Oslo, Norway; 20 Department of Surgical Oncology, Institute of Oncology LjubljanaZaloška 2, 1000, Ljubljana, Slovenia; 21 Department of Surgery, Clinical Sciences, Lund UniversityLund, Sweden; 22 Department of Medical Genetics, University of GlasgowGlasgow, United Kingdom; 23 McGill University and Genome Quebec Innovation CentreMontreal, Canada; 24 Commissariat à l'Energie Atomique (CEA), Institut de Génomique, Centre National de GénotypageEvry, France; 25 Department of Public HealthGlasgow, United Kingdom; 26 Department of Medical GeneticsGlasgow, United Kingdom; 27 Gade Laboratory for Pathology, Department of Clinical Medicine, University of BergenBergen, Norway; 28 Department of Molecular Diagnostics, Institute of Oncology LjubljanaZaloška 2, 1000, Ljubljana, Slovenia; 29 Department of Oncology, Clinical Sciences, Lund UniversitySweden; 30 Department of Cancer Epidemiology, Clinical Sciences, Lund UniversitySweden; 31 Melanoma Unit, Dermatology Department, Hospital Clinic, Institut de Investigacó Biomèdica August Pi Suñe, Universitat de BarcelonaBarcelona, Spain; 32 CIBER de Enfermedades Raras, Instituto de Salud Carlos IIIBarcelona, Spain; 33 Department of Clinical Genetics, Center of Human and Clinical Genetics, Leiden University Medical CenterLeiden, The Netherlands; 34 ServiceXS B.V.Leiden, The Netherlands; 35 Department of Cancer Epidemiology, Moffitt Cancer CenterTampa, FL; 36 Centre for Cancer Genetic Epidemiology, Department of Public Health and Primary Care, Strangeways Research LaboratoryCambridge, United Kingdom; 37 Oncogenomics, QIMR Berghofer Medical Research InstituteBrisbane, QLD, 4029, Australia

**Keywords:** melanoma, fine mapping, penalized regression, heritability, genome-wide signal

## Abstract

At least 17 genomic regions are established as harboring melanoma susceptibility variants, in most instances with genome-wide levels of significance and replication in independent samples. Based on genome-wide single nucleotide polymorphism (SNP) data augmented by imputation to the 1,000 Genomes reference panel, we have fine mapped these regions in over 5,000 individuals with melanoma (mainly from the GenoMEL consortium) and over 7,000 ethnically matched controls. A penalized regression approach was used to discover those SNP markers that most parsimoniously explain the observed association in each genomic region. For the majority of the regions, the signal is best explained by a single SNP, which sometimes, as in the tyrosinase region, is a known functional variant. However in five regions the explanation is more complex. At the *CDKN2A* locus, for example, there is strong evidence that not only multiple SNPs but also multiple genes are involved. Our results illustrate the variability in the biology underlying genome-wide susceptibility loci and make steps toward accounting for some of the “missing heritability.”

**What's new?**

In genome-wide association studies, researchers identify genetic variants that frequently associate with a particular disease, though the variants identified may not contribute to the molecular cause of the disease. This study took a closer look at 17 regions associated with melanoma, fine mapping the regions both in people with melanoma and in healthy controls. Though single SNPs account for the association in some regions, they found that in a few regions, several SNPs – and possibly multiple genes – contributed to the association signal. These findings illustrate the importance of not overlooking the interaction between multiple genetic markers when conducting such studies.

Genome-wide association (GWA) studies have been extremely successful at identifying genomic regions associated with complex diseases and phenotypic traits.^1^ However, studies often do not go beyond reporting the most strongly associated genotyped single nucleotide polymorphism (SNP) and the best candidate gene within the region covered by the association signal. The reported SNP is unlikely to be the causal variant and does not necessarily even identify the relevant gene. Thus the reported SNP is unlikely to characterize the relationship between genotype and phenotype, and hence may not add much to the understanding of disease aetiology.

Although GWA studies involve the genotyping of hundreds of thousands of markers across the genome, a variant not available on the genotyping platform may be more strongly associated with outcome than the single most significant genotyped SNP. Coverage of the region may be greatly improved without extra genotyping by imputation of ungenotyped markers, allowing greater refinement of the association signal. The genetic information gained by imputation may help to identity potential causal variants that are in linkage disequilibrium (LD) with the associated genotyped markers and improve the selection of genes chosen for denser genotyping or sequencing. Furthermore, in some genomic regions multiple markers may better explain the association signal, and multiple variants may be independently associated with the trait. A recent study of the region around the telomerase reverse transcriptase gene *TERT*, within which there are SNPs associated with a number of cancers including melanoma, reported multiple independent SNP associations with both telomere length and breast cancer risk.^2^ For these reasons, disease risk estimates based solely on the single reported SNP may not adequately reflect the contribution of the region to the heritability and aetiology of disease.

Recent GWA studies of melanoma susceptibility, pigmentation-related phenotypes and nevi have led to the discovery and confirmation of a number of genomic regions associated with risk of melanoma.^3^–^8^ Association signals for these regions vary greatly, both in their strength and in the breadth of region showing association. For instance, associated SNPs in the 21q22.3 region (near *MX2*) span <100 kb, whereas associated SNPs in the 20q11.2-q12 region (near *ASIP*) cover more than 1 Mb. This variation may be due largely to differing patterns of LD around a single variant or could be indicative of a more complex arrangement of functional variants. We have previously shown in a melanoma case-control study that fine mapping of an association signal through imputation can help to implicate a gene (*MC1R*) with known functional relevance,^9^ despite the initial association signal spanning a number of candidate genes, the signal here being due to multiple less common loss-of-function variants.

The aim of this study is to refine the association signal in each of the 17 genomic regions previously shown to be associated with melanoma risk using a large case-control dataset.

## Material and Methods

### Study population

Cases for the GenoMEL GWA study of melanoma were preferentially selected to have a family history of melanoma, multiple primary tumors or an onset before 40 years of age, mainly from centers across Europe. In all 2,744 cases and 1,834 controls from Phases 1 or 2 of the study were included in this analysis (see Supporting Information Table 1). An additional 5,857 population-based controls were obtained from the Center National de Génotypage in France or the Wellcome Trust Case Control Consortium (WTCCC) in the UK. Additional cases were obtained from two sources. First, 1,238 cases from the Leeds melanoma cohort were included; this is a population-based study of incident cases diagnosed between September 2000 and December 2006 from a geographically defined area of Yorkshire and the Northern region of the UK.^10^ Second, 1,392 cases were included from the Studies of Epidemiology and Risk Factors in Cancer Heredity (SEARCH) series of population-based studies in Eastern England.^11^ This resulted in a combined sample set of 5,374 cases and 7,691 controls for the analysis after quality control (QC) described below.

### Genotyping

GenoMEL Phase 1 samples were genotyped on the Illumina HumanHap300 BeadChip version 2 duo array and the Illumina HumanCNV370 array. GenoMEL Phase 2 samples were genotyped on Illumina Human610-Quad array. The additional UK cases were genotyped on the Illumina HumanOmniExpressExome BeadChip. The WTCCC samples were genotyped on the Illumina HumanHap 1.2 M array, but only SNPs that were also on either the HumanHap300 or Human610 array were retained for imputation. Samples and SNPs on the HumanOmniExpressExome array were subjected to the same stringent QC as the GenoMEL GWA datasets, previously described in detail.^4^,^5^ Briefly, samples were excluded for any of the following reasons: (*i*) a call-rate of <97% (of the total number of SNPs on the chip); (*ii*) evidence of non-European origin from principal components analysis; (*iii*) sex as ascertained by genotyping not matching reported sex; (*iv*) evidence of first degree relationship or identity with another sample; (*v*) recommendation to be excluded by the WTCCC (for WTCCC samples only). To ensure high quality imputation, very stringent QC measures were applied within each genotyped array; SNPs could therefore be excluded from just a subset of our entire sample. SNPs were excluded for any of the following reasons: (*i*) Hardy–Weinberg equilibrium *p* value <10^−4^ in controls; (*ii*) call-rate <97%; (*iii*) recommendation for exclusion by the WTCCC (for WTCCC samples only); (*iv*) minor allele frequency (MAF) < 0.03.

### Imputation

Imputation was conducted separately on each array using IMPUTEv2^12^ with the 1000 Genomes Phase 1 integrated variant set as reference panel (March 2012 release, excluding SNPs with MAF < 0.001 in CEU European samples). Imputation was constrained to a 2 Mb region (6 Mb for the 20q11.2-q12 region around *ASIP*) centered on the reference SNP. Only those SNPs that were either (*i*) genotyped on all arrays (Type A); (*ii*) imputed with an INFO score ≥0.8 on all arrays (Type B); or (*iii*) imputed with an INFO score ≥0.5 (but not ≥0.8) on all arrays and with a MAF ≥ 0.03 (Type C) were retained for analysis.

### Statistical analysis

A total of 17 regions were analyzed, all of which have been reported to include a SNP associated with melanoma risk, in most instances with a genome-wide level of statistical significance and replication in independent samples (Table 1). For convenience the regions will be referred to by the name of a likely candidate gene.

**Table 1:** Results for 17 previously published melanoma susceptibility regions

			SNP first reported by the reference study				Most significant SNP in this study			
Chromosome	Gene	Reference study	SNP name	Position	*p* value	*R*^2^ for melanoma risk	SNP name	Position	*p* value	*R*^2^ for melanoma risk
1q21.3	*ARNT*	MacGregor et al.^8^	rs7412746	150860471	2.6 × 10^−4^	0.08	rs3768013	150815411	2.9 × 10^−6^	0.13
1q42.12	*PARP1*	MacGregor et al.^8^	rs3219090	226564691	6.4 × 10^−6^	0.12	rs1858550	226608104	1.7 × 10^−7^	0.16
2q33-q34	*CASP8*	Barrett et al.^4^	rs13016963	202162811	9.5 × 10^−7^	0.14	rs2349073	202186986	6.3 × 10^−8^	0.17
5p15.33	*TERT*	Rafnar et al.^17^	rs401681	1322087	4.9 × 10^−11^	0.25	rs2447853	1333077	5.7 × 10^−12^	0.27
5p13.2	*SLC45A2*	Guedj.^18^	rs16891982	33951693	2.2 × 10^−9^	0.20	as reference			
6p25-p23	*IRF4*	Duffy et al.^19^	rs12203592	396321	0.014	0.04	rs9405705	470384	1.5 × 10^−4^	0.08
9p21	*CDKN2A*	Bishop et al.^5^	rs7023329	21816528	8.3 × 10^−14^	0.32	rs869330	21804617	3.8 × 10^−16^	0.38
9p23	*TYRP1*	Duffy et al.^20^	rs2733832	12704725	0.43	0.01	rs72706189	11877260	1.3 × 10^−5^	0.11
11q13	*CCND1*	Barrett et al.^4^	rs1485993	69362414	4.5 × 10^−9^	0.20	rs12422135	69378260	3.5 × 10^−10^	0.22
11q14-q21	*TYR*	Bishop et al.^5^	rs1393350	89011046	5.3 × 10^−13^	0.30	rs1126809	89017961	9.8 × 10^−15^	0.34
11q22-q23	*ATM*	Barrett et al.^4^	rs1801516	108175462	1.8 × 10^−7^	0.16	rs4753835	108145249	1.7 × 10^−7^	0.16
15q13.1	*OCA2*	Amos et al.^3^	rs1129038	28356859	0.081	0.02	rs145720174	28468231	4.8 × 10^−4^	0.07
16q12.2	*FTO*	Iles et al.^21^	rs12596638	54115829	5.1 × 10^−7^	0.14	as reference			
16q24.3	*MC1R*	Bishop et al.^5^	rs258322	89755903	6.8 × 10^−41^	1.02	rs73283859	90062520	3.6 × 10^−52^	1.31
20q11.2-q12	*ASIP*	Bishop et al.^5^	rs2284378	32818707	1.4 × 10^−6^	0.13	rs6059655	32665748	2.1 × 10^−11^	0.26
21q22.3	*MX2*	Barrett et al.^4^	rs45430	42746081	2.9 × 10^−8^	0.18	rs443099	42743327	1.1 × 10^−8^	0.19
22q13.1	*PLA2G6*	Bishop et al.^5^	rs6001027	38545619	3.8 × 10^−7^	0.15	rs3891103	38537159	2.9 × 10^−9^	0.20

Trend test *p* values for association in this study for the SNP reported by the reference study and for the SNP with the strongest signal in this study. The *R*^2^ for percentage of variation explained in melanoma risk is also given from the study. The gene listed is the gene considered to be the likely candidate in the region. Positions are build 37.

For single SNP analyses of association with melanoma, imputed genotypes were analyzed as expected genotype counts based on the posterior probabilities (gene dosage) using logistic regression implemented in SNPTEST2,^18^ assuming an additive model, with geographical region (UK/Netherlands, France, Spain, Scandinavia, Italy, Poland, Israel) as a covariate. We have previously shown that adjusting for region adequately adjusts for population stratification and that including principal components brings no improvement.^4^ No further analysis was conducted for any region where no SNP reached a *p* value<10^−5^ in this analysis. For other regions the SNP-by-SNP analysis was repeated adjusting for the most significant SNP in the region by including this in the logistic regression model.

Each of the regions was narrowed down to the interval covering 500 kb on either side of any SNP with *p* value < 10^−6^ in the initial single SNP unadjusted analysis. Penalized logistic regression is an effective method for the simultaneous analysis of large numbers of correlated variables and was therefore used to jointly analyze all SNPs in each of these narrower intervals. The analysis was carried out using Hyperlasso,^19^ which implements a Bayesian-inspired penalized maximum likelihood approach with a normal-exponential-gamma (NEG) prior. Genotypes were standardized, and geographical region was adjusted for as before. Model parameters were set to control the type-I error at 10^−4^, with the shape parameter fixed at 0.05,^19^ and 100 iterations were run for each region. Each iteration searches for the model with maximum likelihood, but the model may differ between iterations because of the stochastic nature of the order in which variables are considered for inclusion in the model. Each model selected was analyzed further using logistic regression (with no penalization). For interpretation, models were considered to be statistically equivalent if the SNPs included were in complete or very strong LD (based on the correlation coefficient r^2^ between estimated SNP dosages).

For comparison, five regions (*SLC45A2*, *TYR*, *ASIP*, *TERT*, and *CDKN2A*) with different features (see Results) were also analyzed with alternative penalty functions (lasso and elastic net) using the *glmnet* function^20^ in R (version 2.15.2, R Foundation for Statistical Computing, Vienna, Austria, 2012). For this analysis, to aid interpretation LD-based pruning using PLINK^21^ was used to remove markers that were very highly associated (*r*^2^ > 0.95) prior to the penalized regression analysis. The penalty for each term in these analyses is of the form λ (α|β| + (1 − α) β^2^), where β is the coefficient for that term; for lasso, *α* = 1, and for the elastic net we used *α* = 0.5. The multiplier λ was chosen by cross-validation implemented in *glmnet*. As before, geographical region was included in each model and genotypes were standardized.

## Results

About half of all SNPs (genotyped or imputed) were retained for analysis after post-imputation SNP QC (ranging from 37% in *OCA2* to 55% in *PARP1*); most exclusions were on the basis of poor quality of imputation (Supporting Information Table 2).

### SNP-by-SNP analyses

For three regions (*IRF4*, *TYRP1* and *OCA2*), no SNP was associated at *p* < 10^−5^ in these data; these regions were not analyzed further after the initial single SNP analysis. In addition *MC1R* has been analyzed separately,^9^ so results are only presented for the remaining 13 regions.

For two of the regions (*SLC45A2* and *FTO*), the most significantly associated SNP in our data was the same as the top SNP reported in the reference paper. For each of the remaining regions, a more significantly associated SNP was found (Table 1).

**Figure 1:**
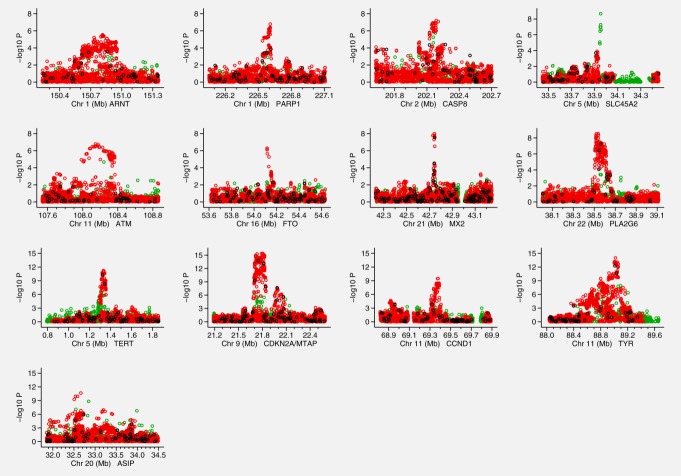
Association signals for the 13 regions analyzed in the fine mapping Manhattan plots displaying the strength of association with melanoma risk (−log_10_ *p*) from the single SNP analysis versus chromosomal position (Mb). The colors indicate the imputation quality: black = fully genotyped (*a*), red = imputed with a minimum INFO score ≥ 0.8 (*b*) and green = imputed with a minimum INFO score ≥0.5 but <0.8 and MAF >3%.

Figure 1 shows Manhattan plots for each of these 13 regions after imputation. The regions near *SLC45A2*, *FTO* and *MX2* all exhibit very narrow association signals, whereas others have signals that encompass several genes, the widest of these being the *ASIP* region covering several megabases. When adjusting for the most significant SNP, the *TERT*, *CDKN2A* and *CCND1* regions have a clear secondary signal reaching at least 10^−5^ (Supporting Information Fig. 1).

### Hyperlasso analyses

#### Single variant regions

In the Hyperlasso analyses, the effect on disease risk was best described by a single-SNP model for 8 regions (*ARNT*, *PARP1*, *CASP8*, *SLC45A2*, *TYR*, *ATM*, *FTO* and *MX2*); for each of these regions a single-SNP model was selected in at least 89% of the iterations. For the remaining few iterations a 2-SNP model was selected. When a single-SNP model was selected, the SNP was almost always either the most significant SNP from the single SNP analysis (see Table 1) or one in strong LD with it (almost always *r*^2^ > 0.9, Supporting Information Table 3).

For the *ARNT* region, the SNPs selected in the model were located within the *ARNT* gene in 90% of the iterations. The most strongly associated SNP in the region is rs3768013, which was selected in 13 of the 100 iterations. Another SNP, rs7514004, in almost complete LD with this (*r*^2^ = 0.99) was selected slightly more frequently (16 times), and 8 other SNPs were selected in different iterations, all also in strong LD (*r*^2^ ≥ 0.98 for 7 of them, *r*^2^ = 0.92 for one). For the *PARP1* region only 6 out of 100 iterations selected a SNP actually located in the *PARP1* gene; otherwise a SNP located near the 5′ end was selected. Across all iterations, 28 different SNPs were selected, but all were in very strong LD with the top SNP rs1858550 (*r*^2^ ≥ 0.97). Similarly, for the *CASP8* region, only 17% of the iterations converging to a single-SNP model selected a SNP in *CASP8*, with the majority selecting a SNP in the neighboring gene *AL2CR12*. The top SNP rs2349073 was selected most frequently (13 iterations), but others were similarly frequent and all were in strong LD (*r*^2^ ≥ 0.94). Only one SNP was selected, in all 100 iterations, for the *SLC45A2* region, this being the previously reported missense SNP rs16891982. This SNP was also selected using either the lasso or elastic net penalty. Similarly, the missense SNP rs1126809 was selected in 44% of the single-SNP models for the *TYR* region; in most other iterations an alternate SNP in LD with this was selected (*r*^2^ ≥ 0.93 in all but 9 iterations, *r*^2^ ≥ 0.89 otherwise). Using the alternative penalty functions also resulted in selection of rs1126809. For the *ATM* region, in 82 of the 100 iterations a SNP in the *ATM* gene itself was selected; although 15 different SNPs were selected, all were in almost complete LD with the top SNP rs4753835 (*r*^2^ ≥ 0.98). Only three distinct SNPs were selected for the *FTO* region, all in the *FTO* gene; one was the top SNP rs12596638, and the other two were in almost perfect LD with this (*r*^2^ ≥ 0.99). In the *MX2* region the top SNP rs443099, or one of five other SNPs within the *MX2* gene in almost perfect LD (*r*^2^ ≥ 0.98), was selected in 81% of the iterations converging on a single-SNP model. For all but one of the remaining iterations, rs390789, a SNP in weaker LD (*r*^2^ = 0.86) and not in *MX2*, was selected.

#### Possible multiple variant region

For the *PLA2G6* region a single-SNP model was selected in 66 iterations (Supporting Information Table 4), the single SNP being either the top SNP rs3891103 or one in moderate to strong LD with this (*r*^2^ ≥ 0.82). In other iterations 2-SNP models were selected involving SNPs both of which were reasonably strongly associated with rs3891103 (*r*^2^ ≥ 0.59, usually much higher). In most models, both SNPs were located in the *PLA2G6* gene. The results are not as clear-cut as for the above single-SNP regions; a possible explanation is that a single causal SNP exists in the region that is not in very strong LD with any single genotyped or imputed SNP.

#### Regions showing evidence of multiple independent variants

**Figure 2:**
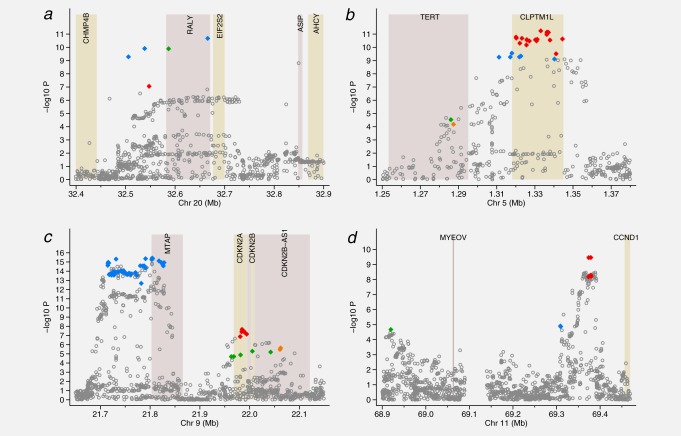
Regions showing evidence of multiple independent signals Manhattan plots displaying the strength of association with melanoma risk (−log_10_ *p*) from the single SNP analysis versus chromosomal position (Mb). The colored diamonds indicate the SNPs selected by Hyperlasso. Those of the same color are in strong LD with *r*^2^ ≥ 0.80 and correspond to the colored blocks in Supporting Information Tables 5–8. Shaded regions show the position of the genes in the region. (*a*) *ASIP* (*b*) *TERT* (*c*) *CDKN2A* (*d*) *CCND1*.

Hyperlasso gave a variety of models for the *ASIP* gene (Table 2, Supporting Information Table 5). Most of these reduce to two different 2-SNP models occurring with similar frequency. Both models include rs6059655 in *RALY* (or a SNP in almost complete LD, *r*^2^ ≥ 0.98, blue diamonds in Fig. 2*a*), the other SNP being either rs6088372 also in *RALY* (green diamond) or rs74325991 (red diamond) which is not located in a gene. Using the alternative penalties, the 2-SNP model including rs6059655 and rs6088372 was selected.

**Table 2:** SNPs selected in models for the regions showing evidence for multiple independent associations

						Single SNP result			Logistic regression of multiple variant models		
Region	SNP name	Position	Mapped gene	Allele	Allele frequency	OR	*p* value	*r*^2^ with top SNP	OR	*p* value	*R*^2^ for melanoma risk
*TERT*	rs7705526	1285974	*TERT*	A	0.332	1.13	2.9 × 10^−5^	0.09	1.09	0.026	0.46
	rs2736099	1287340	*TERT*	A	0.374	1.12	6.6 × 10^−5^	0.14	1.09	0.025	
	rs1801075	1317949	*intergenic*	C	0.172	1.23	2.7 × 10^−10^	0.51	1.08	0.050	
	rs2447853	1333077	*CLPTM1L*	G	0.468	1.20	5.7 × 10^−12^	Top SNP	1.18	1.3 × 10^−7^	
*CDKN2A*	rs869330	21804617	*MTAP*	G	0.513	0.81	3.9 × 10^−16^	Top SNP	0.81	8.0 × 10^−16^	0.65
	rs3088440	21968159	*CDKN2A*	A	0.089	1.21	2.0 × 10^−5^	0.03	1.13	0.014	
	rs3731204	21984661	*CDKN2A*	C	0.148	0.81	2.2 × 10^−8^	0.03	0.84	8.1 × 10^−6^	
	rs1011970	22062134	*CDKN2B-AS1*	T	0.166	1.17	2.3 × 10^−6^	0.02	1.09	0.033	
*CCND1*	rs2290419	68919649	*intergenic*	G	0.057	0.78	2.1 × 10^−5^	0.03	0.76	7.2 × 10^−6^	0.37
	rs623110	69308897	*intergenic*	T	0.314	1.13	1.3 × 10^−5^	0.35	1.07	0.015	
	rs12422135	69378736	*intergenic*	A	0.409	1.18	3.5 × 10^−10^	Top SNP	1.15	3.1 × 10^−7^	
*ASIP*	rs74325991	32547380	*intergenic*	G	0.490	1.18	8.8 × 10^−8^	0.38	1.11	0.0025	0.31
	rs6059655	32665748	*RALY*	A	0.086	1.33	2.1 × 10^−11^	Top SNP	1.26	4.6 × 10^−7^	
											

For each region the model with the greatest number of SNPs in shown after 100 iterations of Hyperlasso. Two different 2-SNP models occurred for ASIP, both include rs6059655 with either rs74325991 (presented here) or rs6088372 (not shown). The ORs (odd ratios) for the stated allele and *p* values are presented for the results from the single SNP analysis and when including all listed SNPs at that locus. The LD (*r*^2^) with the most significant SNP in this study (Top SNP) is estimated from the correlation coefficient. The *R*^2^ for percentage of variation explained in melanoma risk is given for including all listed SNPs at that locus.

For all three of the regions (*TERT*, *CDKN2A* and *CCND1*) that showed strong evidence of further association when adjusting for the top SNP (Supporting Information Fig. 1), Hyperlasso selected multiple variant models in all iterations.

Either 3- or 4-SNP models were selected to explain the signal in the *TERT* region (Table 2, Supporting Information Table 6). When taking LD into account, these reduced to two distinct 3-SNP models (selected in 33 and 12% of iterations) and one 4-SNP model (55%). The most commonly selected 3-SNP model includes 2 SNPs in the *TERT* gene (rs7705526 and rs2736099, *r*^2^ = 0.60, *D*′ = 0.60 between them, green and orange diamonds in Fig. 2*b*) and the top single SNP rs2447853 (or one in almost complete LD with this) in the neighboring *CLPTM1L* gene (red diamonds in Fig. 2*b*). The LD between rs2447853 and the 2 SNPs in *TERT* was *r*^2^ = 0.09, *D*′ = 0.11 for rs7705526 and *r*^2^ = 0.14, *D*′ = 0.18 for rs2736099. When regressing melanoma case/control status on these 3 SNPs, the signal at *CLPTM1L* becomes stronger than in the single-SNP analysis (*p* = 5.5 × 10^−14^). The 4-SNP model was equivalent to this 3-SNP model, but with an extra SNP, rs1801075, which lies between *TERT* and *CLPTM1L*, or a SNP in strong LD with this, which includes SNPs within *CLPTM1L* (blue diamonds in Fig. 2*b*). This 4-SNP model was selected using the alternative penalty functions. The imputation quality was noticeably poor around the *TERT* gene; only 29% of variants in the gene (from 1,000 Genomes) are included in the analysis, compared with 50% across all regions, and 64 of the 78 SNPs that are included are relatively poorly imputed (Type C) SNPs.

The signal in the *CDKN2A* region is explained in most iterations by a particular 3-SNP (31 iterations) or 4-SNP model (54 iterations) (Table 2, Supporting Information Table 7). All these models included the top SNP rs869330, which is in *MTAP*, or a SNP in strong LD with this (blue diamonds in Fig. 2*c*). There was almost no change in the OR and *p* value for this SNP when adding the other 2 SNPs in the 3-SNP model in a logistic regression analysis. The other 2 SNPs in the model also gave reasonably strong signals in the multiple logistic regression analysis. These were located in *CDKN2B-AS1* or *CDKN2A* (rs3088440 or a SNP in strong LD with this, *p* < 10^−4^, green diamonds in Fig. 2*c*) and *CDKN2A* (rs3731204 or a SNP in LD with this, *p* < 10^−5^, red diamonds in Fig. 2*c*). The LD between rs869330 and the other 2 SNPs was *r*^2^ = 0.03 for both rs3088440 and rs3731204 (*D*′ < 0.07). The LD between rs3088440 and rs3731204 was *r*^2^ = 0.12, although *D*′ = 0.94: the minor allele for rs3088440 hardly ever occurs with the minor allele of rs3731204. The 4-SNP model was equivalent to the 3-SNP model with an additional SNP, rs1011970 or a proxy, in *CDKN2A-AS1* (orange diamonds in Fig. 2*c*). The remaining 15 iterations converged on 3-, 4- or 5-SNP models, which were similar (usually including all the SNPs from the 3-SNP model). Using the lasso penalty, a 6-SNP model was selected, including the 4 SNPs in the above 4-SNP model, plus an additional 2 SNPs from among those highly correlated with the top SNP rs869330 (blue diamonds in Fig. 2*c*).

The most frequent model selected for the *CCND1* region (65 iterations) was a 2-SNP model with neither SNP located in a gene (Table 2, Supporting Information Table 8, Fig. 2*d*). The models included the top SNP rs12422135 (*p* < 10^−9^) or a proxy, all of which were around 80 kb from *CCND1* (red diamonds in Fig. 2*d*). The second SNP in the model was rs2290419 (*p* < 10^−5^) located distal to *MYEOV* (green diamond in Fig. 2*d*). These SNPs are not in LD with one another (*r*^2^ = 0.03, *D*′ = 0.12). In 20 iterations a third SNP was also selected (rs623110 or rs486564, *r*^2^ = 1.0, blue diamond in Fig. 2*d*). In the remaining 15 iterations, alternative 3- or 4-SNP models were selected, all of which were equivalent to one of these two models plus one additional SNP.

#### Improvements in explanatory power

For each region the percentage of variance in melanoma risk explained by the reported SNP and the top SNP from this study is shown in Table 1. If we assume the SNPs contribute additively to risk, the 13 SNPs studied in detail are estimated to explain 2.4% of the variance in risk based on the reported SNP, rising to 2.8% if we use the top single SNP based on imputation in this study. Hence the improvement is modest (17%), and will be partly driven by over-fitting; the largest single improvement is for *ASIP*, where the estimate doubles from 0.13 to 0.26%. For the 3 clearly more complex regions, the percentage of variance explained by the models in Table 2 compared with the best single-SNP model increases by ∼70% (70% for *TERT*, 71% for *CDKN2A* and 68% for *CCND1*).

## Discussion

We have refined the association signals for regions that have been previously associated with melanoma, using a pragmatic statistical approach that includes adjusted analyses and penalized logistic regression. We have shown that the complexity of the association signal within a specific genomic region ranges from those regions best explained by a single variant to those that can only be explained by 3 or 4 variants. The evidence for multiple independent signals is strong: in three regions there is a secondary signal reaching *p* < 10^−5^ after conditioning on the most significant SNP, equivalent to a Bonferroni correction for 5,000 independent tests in the region, and these results are borne out by the Hyperlasso analysis. It is possible that even independent signals represent a haplotypic effect, although we saw little evidence of haplotypic effects from the SNPs in the multiple variant regions. It is becoming increasingly clear that multiple independent causal variants may contribute to disease susceptibility at a single locus.^2^ Despite this, statistical approaches are sometimes applied to fine mapping that presuppose the existence of a single causal variant in a region.^22^,^23^

We found strong evidence that a single SNP explains the association signal in 8 of the 13 regions analyzed here: *ARNT*, *PARP1*, *CASP8*, *SLC45A2*, *TYR*, *ATM*, *FTO* and *MX2*. For two of the regions (*SLC45A2* and *TYR*), the likely casual variants are known. The SNP rs16891682 in *SLC45A2* was the only SNP selected by Hyperlasso and was also detected using other penalty functions; this SNP would likely be identified by any reasonable method. The SNP rs1126809 in *TYR* was selected using lasso/elastic net and about half of the time using the Hyperlasso method; the remaining iterations all converged on a single-SNP model where the SNP selected was in reasonably strong LD with rs1126809 (*r*^2^ ≥ 0.89). For the other single-variant regions identified, although no one SNP was selected much more frequently than the others, this was largely due to the inability of any statistical method to distinguish between almost perfectly correlated variables. The evidence from analysis of these regions suggests that either the most strongly associated SNP or one in very strong LD with it is the most likely explanation of the association signal; bioinformatic analysis of this relatively small set of SNPs can now be used to suggest the most promising candidates for functional investigation.

More complex models were clearly needed to explain the signals for the regions near *TERT*, *CDKN2A* and *CCND1*. Interestingly all three of these regions have previously been reported as harboring multiple risk variants for other diseases or traits. Independent associations have previously been reported in *TERT* for breast cancer and telomere length.^2^ The telomere associations partially concur with our model for melanoma; the SNPs associated with telomere length are rs7705526, the SNP indicated by a green diamond in Figure 2*b*, and rs2736108, which is only nominally associated with melanoma in our analysis (*p* = 0.008). Our reported second SNP in *TERT*, rs2736099 (orange diamond), is in only moderate LD with rs2736108 (*r*^2^ = 0.49, *D*′ = 0.60), although both are strongly associated with telomere length in univariate analysis (*p* < 10^−5^ in Bojesen et al.^2^). The *TERT* SNP alleles associated with longer telomeres are associated with higher risk of melanoma. In addition our most significant SNP in the region was in the neighboring *CLPTMIL* gene; SNPs in this gene show no clear association with either telomere length or breast cancer.

We found 3 strong independent signals in the 9p21 region, the strongest being in *MTAP*, with secondary peaks in the region containing *CDKN2A* and *CDKN2B-AS1*. The variant rs10811656, associated with coronary artery disease (CAD),^24^,^25^ is peripheral to this region (at 22.12 Mb in the *CDKN2B-AS1* locus, Fig. 2*c*). The interval around rs10811656 has been studied using chromatin conformation capture in human vascular endothelial cells^26^ and shown to physically interact with both the *CDKN2A/B* locus and *MTAP*. This complex region is clearly of major significance in a number of diseases.

French et al.^27^ found evidence for 3 distinct signals in the *CCND1* region in relation to oestrogen-receptor-positive breast cancer. Although different SNPs to ours were identified, their signals were between 69.32 and 69.38 Mb (Build 37), which is roughly the region spanned by two of the three signals in our model (blue and red diamonds in Fig. 2*d*). Although this region is itself intergenic, on the basis of functional studies these authors conclude that *CCND1* is the likely target gene for the variants identified.

Here we have employed penalized regression with a NEG prior to fine map these loci. This choice of prior was motivated by its sharp peak at zero, which shrinks the regression coefficients strongly when they are close to zero, leading to sparse models. In a comparison of penalized logistic regression methods with different penalties, single locus analysis and stepwise regression, Ayers and Cordell^28^ showed that the NEG gave the best overall performance and did not suffer from limitations on the number of markers being considered. Reassuringly we found broadly similar results when using a lasso or elastic net penalty function, although where there were differences these latter methods seemed to favor models with larger numbers of SNPs, which were then not significant in the full model using classical logistic regression.

A major limitation of statistical fine mapping based on imputation is that about half of all possible variants (as identified by 1,000 genomes) are dropped because they cannot be reliably imputed, at least with the density of genotyping used in our study and after strict QC. There is therefore a need to more densely genotype, or preferably sequence, parts of these regions to follow up these analyses. The analysis presented here helps to prioritize which of the associated loci require further investigation and, within these, to narrow down the regions to be sequenced.

We found a substantial (70%) improvement in the proportion of variance in melanoma risk explained by multiple SNP models compared with single SNPs in selected regions, although overall the proportion of variance explained by all loci is only modestly increased. This has been explored in other traits,^29^ showing an average increase of 17% in the proportion of variance explained using regression-based analysis of jointly significant markers compared with single variants at each locus.

Statistical fine mapping does not in itself identify the causal SNP(s) but it does take us closer to achieving this goal by narrowing down the number of SNPs to be considered for further investigation. In all but the very simplest regions (*SLC45A2* and *TYR*), where coding variants explaining the signal have been previously identified, fine mapping must be followed up using bioinformatics and experimental approaches. Methods to identify and follow up non-coding functional variants have recently been reviewed,^30^ with suggestions of bioinformatics database searches, application of *in silico* tools and a range of molecular experimental techniques that can take the process forward to identify the causal mechanisms.
