# Supplementary material for: Fine mapping of genetic susceptibility loci for melanoma reveals a mixture of single variant and multiple variant regions
Source: Int J Cancer. 2014 Jul 31;136(6):1351–60. doi: 10.1002/ijc.29099 (PMC4328144; doi:10.1002/ijc.29099)
Supplement: Supplementary file 1 — Supplementary Information [file ijc0136-1351-sd1.docx]

**GenoMEL membership:**

*Australian Melanoma Family Study:* Graham J. Mann, John L. Hopper, Joanne F. Aitken, Bruce K. Armstrong, Graham G. Giles, Elizabeth Holland, Richard F. Kefford, Anne Cust, Mark Jenkins, Helen Schmid.

*Barcelona:* Paula Aguilera, Celia Badenas, Cristina Carrera, Francisco Cuellar, Daniel Gabriel, Estefania Martinez, Melinda Gonzalez, Pablo Iglesias, Josep Malvehy, Rosa Marti-Laborda, Montse Mila, Zighe Ogbah, Joan-Anton Puig Butille, Susana Puig and Other members of the Melanoma Unit: Llúcia Alós, Ana Arance, Pedro Arguís, Antonio Campo, Teresa Castel, Carlos Conill, Jose Palou, Ramon Rull, Marcelo Sánchez, Sergi Vidal-Sicart, Antonio Vilalta, Ramon Vilella.

*Brisbane: The Queensland study of Melanoma: Environmental and Genetic Associations (Q- MEGA) Principal Investigators are:* Nicholas G. Martin, Grant W. Montgomery, David Duffy, David Whiteman, Stuart MacGregor, Nicholas K. Hayward. *The Australian Cancer Study (ACS) Principal Investigators are:* David Whiteman, Penny Webb, Adele Green, Peter Parsons, David Purdie, Nicholas Hayward.

*Emilia-Romagna:* Maria Teresa Landi, Donato Calista, Giorgio Landi, Paola Minghetti, Fabio Arcangeli, Pier Alberto Bertazzi.

*Genoa: Department of Internal Medicine and Medical Specialties, Laboratory of Genetics of Rare Hereditary Cancers, University of Genoa/San Martino-IST Research Hospital:* Giovanna Bianchi Scarrà, Paola Ghiorzo, Lorenza Pastorino, William Bruno, Sabina Nasti, Linda Battistuzzi, Paola Origone, Virginia Andreotti. *Medical Oncology Unit, San Martino-IST Research Hospital:* Paola Queirolo.

*Glasgow:* Rona Mackie, Julie Lang.

*Leeds:* Julia A Newton Bishop, Paul Affleck, Jennifer H Barrett, D Timothy Bishop, Jane Harrison, Mark M Iles, Juliette Randerson-Moor, Mark Harland, John C Taylor, Linda Whittaker, Kairen Kukalizch, Susan Leake, Birute Karpavicius, Sue Haynes, Tricia Mack, May Chan, Yvonne Taylor, John Davies, Paul King.

*Leiden: Department of Dermatology, Leiden University Medical Centre:* Nelleke A Gruis, Frans A van Nieuwpoort, Coby Out, Clasine van der Drift, Wilma Bergman, Nicole Kukutsch, Jan Nico Bouwes Bavinck. *Department of Clinical Genetics, Centre of Human and Clinical Genetics, Leiden University Medical Centre:* Bert Bakker, Nienke van der Stoep, Jeanet ter Huurne. *Department of Dermatology, HAGA Hospital, The Hague:* Han van der Rhee. *Department of Dermatology, Reinier de Graaf Groep, Delft:* Marcel Bekkenk. *Department of Dermatology, Sint Franciscus Gasthuis, Rotterdam:* Dyon Snels, Marinus van Praag. *Department of Dermatology, Ghent University Hospital, Ghent, Belgium:* Lieve Brochez and colleagues. *Department of Dermatology, St. Radboud University Medical Centre, Nijmegen:* Rianne Gerritsen and colleagues. *Department of Dermatology, Rijnland Hospital, Leiderdorp:* Marianne Crijns and colleagues. *Dutch patient organisation, Stichting Melanoom, Purmerend. The Netherlands Foundation for the detection of Hereditary Tumors, Leiden:* Hans Vasen. *ServiceXS:* Wilbert van Workum, Bart Janssen, Marjolein Janssen and Suzanne Mulder

*Lund:* Lund Melanoma Study Group: Håkan Olsson, Christian Ingvar, Göran Jönsson, Åke Borg, Anna Måsbäck, Lotta Lundgren, Katja Baeckenhorn, Kari Nielsen, Anita Schmidt Casslén.

*Norway: Oslo University Hospital:* Per Helsing, Per Arne Andresen, Helge Rootwelt. *University of Bergen:* Lars A. Akslen, Anders Molven.

*Paris:* Florence Demenais, Marie-Françoise Avril, Brigitte Bressac-de Paillerets, Valérie Chaudru, Nicolas Chateigner, Eve Corda, Patricia Jeannin, Fabienne Lesueur, Mahaut de Lichy, Eve Maubec, Hamida Mohamdi and the French Family Study Group including the following Oncogeneticists and Dermatologists: Pascale Andry- Benzaquen, Bertrand Bachollet, Frédéric Bérard, Pascaline Berthet, Françoise Boitier, Valérie Bonadona, Jean-Louis Bonafé, Jean-Marie Bonnetblanc, Frédéric Cambazard, Olivier Caron, Frédéric Caux, Jacqueline Chevrant-Breton, Agnès Chompret (deceased), Stéphane Dalle, Liliane Demange, Olivier Dereure, Martin-Xavier Doré, Marie-Sylvie Doutre, Catherine Dugast, Laurence Faivre, Florent Grange, Philippe Humbert, Pascal Joly, Delphine Kerob, Christine Lasset, Marie Thérèse Leccia, Gilbert Lenoir, Dominique Leroux, Julien Levang, Dan Lipsker, Sandrine Mansard, Ludovic Martin, Tanguy Martin-Denavit, Christine Mateus, Jean-Loïc Michel, Patrice Morel, Laurence Olivier-Faivre, Jean-Luc Perrot, Caroline Robert, Sandra Ronger- Savle, Bruno Sassolas, Pierre Souteyrand, Dominique Stoppa-Lyonnet, Luc Thomas, Pierre Vabres, Eva Wierzbicka.

*Philadelphia:* David Elder, Peter Kanetsky, Jillian Knorr, Michael Ming, Nandita Mitra, Althea Ruffin, Patricia Van Belle

*Poland:* Tadeusz Dębniak, Jan Lubiński, Aneta Mirecka, Sławomir Ertmański. *Slovenia:* Srdjan Novakovic, Marko Hocevar, Barbara Peric, Petra Cerkovnik. *Stockholm:* Veronica Höiom, Johan Hansson. *Sydney:* Graham J. Mann, Richard F. Kefford, Helen Schmid, Elizabeth A. Holland

*Tel Aviv:* Esther Azizi, Gilli Galore-Haskel, Eitan Friedman, Orna Baron-Epel, Alon Scope, Felix Pavlotsky, Emanuel Yakobson, Irit Cohen-Manheim, Yael Laitman, Roni Milgrom, Iris Shimoni, Evgeniya Kozlovaa.
