# Supplementary figures and images for: Fine mapping of genetic susceptibility loci for melanoma reveals a mixture of single variant and multiple variant regions
Source: Int J Cancer. 2014 Jul 31;136(6):1351–60. doi: 10.1002/ijc.29099 (PMC4328144; doi:10.1002/ijc.29099)

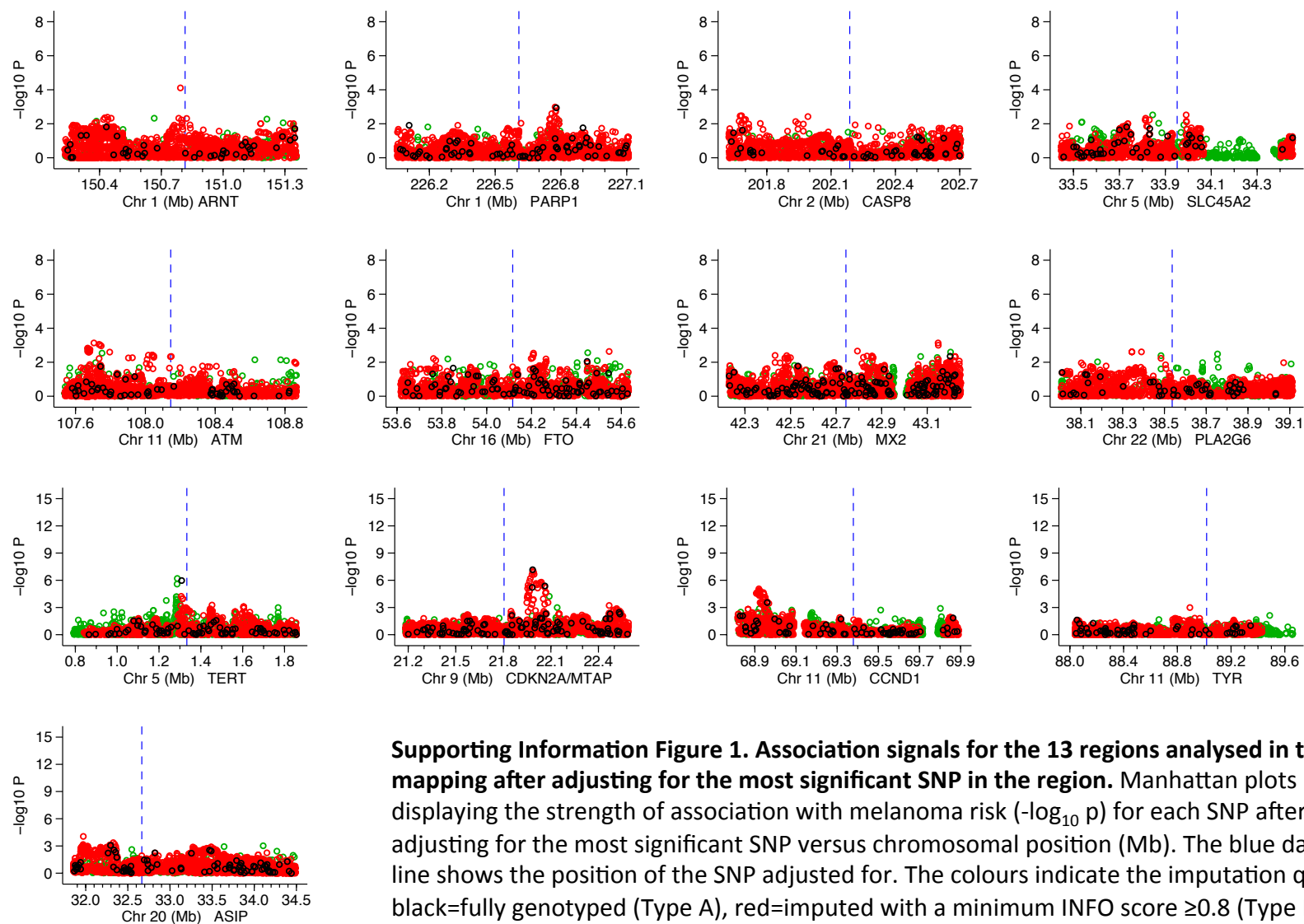

Supplement: Supplementary file 2 — Supplementary Figure 1 [file ijc0136-1351-sd2.pdf]
